# Sternum hypoplasia diagnosed during sternotomy

**DOI:** 10.1186/1749-8090-8-S1-P59

**Published:** 2013-09-11

**Authors:** I Duvan, S Ates, M Kurtoglu, BE Onuk, P Sungur, IS Karacan

**Affiliations:** 1 Department of Cardiovascular Surgery, Güven Hospital, Ankara, Turkey

## Background

The sternum (breastbone) is a long, flattened bone, laying vertically and constituting the middle part of the anterior wall of the thorax. It is clinically important for us, because median sternotomy is the most common surgical approach used in cardiac surgery. It has three parts named from above to downward; manubrium, corpus and xiphoid. Its average length in the adult is about 17 cm and is rather shorter, narrower and thinner in the female than male.

## Methods

A 48 year old diabetic female patient admitted to our hospital suffering from chest pain and her effort test was (+) too. Coronary angiography demonstrated atherosclerotic lesions in LAD and diagonal arteries. CABG was recommended to these vessels via LIMA sequentially. She was diagnosed to have a sternal hypoplasia incidentally during sternotomy. It was only 6 cm long and there was only corpus sterni without the manubrium and xiphoid processes. LIMA was harvested without any problem and its length was normal. Sternal closure was finished by two figure-of-8 sutures via stainless steel wires after OPCABGx2 was performed uneventfully.

## Results

Operation time was 45 minutes. Intubation period was 11, ICU stay was 22 hours. Sternum was protected against dehiscence by the help of a corset and the patient was discharged on the 4th day without any problems about the operation.

## Conclusions

We presented a case of sternal hypoplasia diagnosed during sternotomy for OPCABG.

